# Teacher Anxiety Program for Elementary Students (TAPES): intervention development and proposed randomized controlled trial

**DOI:** 10.1186/s13063-019-3863-9

**Published:** 2019-12-30

**Authors:** Golda S. Ginsburg, Jeffrey E. Pella, Kate Piselli, Grace Chan

**Affiliations:** 1 University of Connecticut School of Medicine, 65 Kane Street Room 2033, West Hartford, CT 06119 USA; 2 University of Connecticut School of Medicine Department of Psychiatry, 263 Farmington Avenue, Farmington, CT 06030-2103 USA

**Keywords:** School-based, Effectiveness research, Anxiety disorders, Randomized controlled trial, Elementary school teachers

## Abstract

**Background:**

Excessive student anxiety is a common problem that severely impairs short- and long-term academic functioning and increases teacher burden. Reducing student anxiety has been associated with improvement in educational functioning. Because anxiety manifests daily in the classroom, teachers are in an ideal position to identify and help students manage their anxiety. Unfortunately, teachers lack the knowledge and skills to support the learning of students with excessive anxiety. The Teacher Anxiety Program for Elementary Students (TAPES), a novel teacher-administered school-home collaborative intervention, was designed to address this gap.

**Methods:**

This manuscript describes the protocol for developing and evaluating TAPES. Specifically, we present a description of: (1) the intervention and theoretical model; and (2) methods for the proposed randomized controlled trial comparing TAPES to a standard professional development seminar focused on reducing student anxiety.

**Discussion:**

Primary aims examine the impact of the TAPES training on teacher knowledge and skill. Secondary aims examine the impact of TAPES on student outcomes. Exploratory aims will examine mediators based on our proposed theory of change. If effective, TAPES has the potential to directly benefit teachers (improving skills) and students (reducing anxiety and improving functioning).

**Trial registration:**

ClinicalTrials.gov, NCT03899948. Registered on 28 March 2019.

## Introduction

Excessive anxiety is among the most common forms of pediatric psychopathology and severely impairs academic functioning [1, 2]. Students with excessive anxiety present challenges to teachers who require specialized skills to manage students’ anxiety-related social, emotional, behavioral, and educational issues in the classroom [3–6]. For instance, children with separation anxiety, which is characterized by excessive distress upon separating from parent(s), can experience intense symptoms of anxiety during morning drop-off time. Elementary school teachers often assist these children, peeling them away from their caregiver(s), helping them calm down, and ensuring that they stay in their classroom and engage in classroom activities. These children might request to call and check-in with their parent several times during the day, interrupting instruction and interfering with their own and others’ learning. For students with generalized anxiety, academic demands often trigger excessive and persistent worries about performance and perfectionism. These students are often preoccupied with fears of making mistakes, failing, and disappointing their teachers, which negatively impair their classroom behavior, seeking reassurance often from their teacher. Finally, students with social anxiety, characterized by excessive fears of embarrassing oneself or being criticized often avoid answering or asking questions in class, approaching teachers for help, and working on group projects.

Although a teacher’s primary duty is to educate, the role (and need) of teachers has broadened to include understanding and even intervening to reduce mental health symptoms, including anxiety. However, the vast majority of teachers never receive any evidenced-based training for identifying or assisting students with excessive anxiety [7]. Fortunately, meta-analyses indicate that with adequate training and coaching, teachers can effectively deliver “universal” classroom-based social–emotional curricula with numerous positive effects on student outcomes [8–15].

The potential benefits of training teachers to reduce student anxiety is also informed by emerging evidence that suggests reducing anxiety improves school performance [16, 17]. Data from randomized controlled studies of child anxiety treatments delivered by community or school clinicians have shown that decreases in anxiety are associated with increases in Grade Point Average (GPA) and the normalization of GPAs among test-anxious youth compared to their non-test-anxious peers [16].

To address the need for teacher-led interventions for student anxiety, a three-year study, funded by the Department of Education (R324A170071), is being conducted in three stages. Stages one and two focus on the development of the Teacher Anxiety Program for Elementary Students (TAPES), a novel teacher-led school–home program for anxious students and ensuring its feasibility and acceptability through iterative open trials. Stage three, described below, is a planned randomized controlled effectiveness trial comparing the impact of TAPES on teacher and student outcomes relative to a typical professional development seminar on student anxiety (referred to as Teacher Anxiety Training [TAT]). The three stages of this study will pursue the following aims:

- Aim 1: develop TAPES and assess its usability, acceptability, and feasibility;
- Aim 2: determine whether teachers can implement TAPES with high fidelity and quality;
- Aim 3: examine the impact of TAPES on teacher knowledge and skills related to anxiety-reduction strategies;
- Aim 4: examine the impact of TAPES on student anxiety and academic functioning.

## Materials and methods

### Participants

#### Teacher participants

A total of 40 volunteer elementary school teachers are expected to participate. The racial/ethnic make-up of teachers will likely reflect teachers in the state of Connecticut: 3.7% Black or African American; 3.7% Hispanic/Latino; 91.0% Caucasian; 1.1% Asian; and 0.1% two or more races [18]. Teachers of all races/ethnicities may be eligible to participate. All participants must be a regular or special education elementary teacher for the Connecticut public school system. There are no other inclusion/exclusion criteria to enhance the generalizability of the study findings.

#### Student participants

A total of 60 elementary school students will participate in the randomized controlled trial (RCT) and will reflect the socioeconomic and racial/ethnic background of students in Connecticut (12.8% African American, 24.8% Hispanic/Latino, 53.6% Caucasian, 5.1% Asian, 3.3% two or more races; 36.9% receive free/reduced priced meals [19, 20]). Children of all races/ethnicities may be eligible to participate and proportions will depend on the sample of volunteers.

#### Student inclusion/exclusion criteria

Inclusion/exclusion criteria were crafted to maximize the generalizability of findings. All students must: (1) attend elementary school in Connecticut (i.e. ages 5–12 inclusive); and (2) have elevated anxiety symptoms (i.e. a Spence Child Anxiety Scale [SCAS] [21], T score ≥ 60 based on parent and/or child report and/or a Clinician Severity Rating of ≥ 3 for a DSM-5 anxiety diagnosis on the Anxiety Disorders Interview Schedule [ADIS] [22]). Students will be excluded if they have a medical or psychiatric condition contraindicating study participation (based on clinical interview such as recent suicidality). Students may be receiving concomitant mental health interventions. Ambiguous cases will be decided by the principal investigators (PIs), evaluator, and teacher.

### Study conditions

#### TAPES

The core components of TAPES are based on “common elements” of cognitive behavioral therapy (CBT) [23–26]. TAPES is delivered by the teacher with each individual family at school. TAPES includes five 30-min conjoint meetings with the teacher, a student, and his/her parent(s) over an eight-week period (the first meeting is with parent and teacher only). The 30-min meeting length is based on previous school–home intervention models [27]. These meetings can be supplemented by phone and email contact between teachers and parents as needed. The importance of close interaction between school and home settings has been emphasized for decades, and psychosocial interventions that incorporate both home and school components have been successful in improving academic and behavioral outcomes [28]. Moreover, data from school–home intervention studies reveal that teacher-reported improvements in relationships with parents mediate intervention effects on positive changes in child outcomes. The section below describes the content of the TAPES meetings and a proposed theory of teacher and child behavior change. All conjoint meetings will be audio-recorded for review of fidelity and quality of implementation.

##### Meeting 1: recognizing anxiety (parent only)

Teachers orient parents to TAPES, including the rationale, goals, and potential benefits of the program. Guided by the TAPES manual, teachers provide parents with anxiety-related psychoeducation, including the cognitive behavioral model [29]. Additionally, teachers provide an overview of the cognitive behavioral model of anxiety reduction to presage the skills to be taught to the student. An emphasis is placed on conducting “exposure” or engaging in brave behaviors and initial use of positive reinforcement to reward “brave” (i.e. non-anxious or non-avoidant) behavior. Teachers and parents review their own behaviors that increase student anxiety and both plan to modify/decrease these behaviors (e.g. accommodation of fear/anxious avoidance, hostility, over-control) and increase behaviors that can reduce anxiety (e.g. warmth, autonomy promotion). These modifications are also designed to improve the quality of the teacher–student relationship reinforcing they are both on the same team and each have a unique role in assisting the student.

##### Meeting 2: recognizing anxiety and learning relaxation skills

In this meeting, the teacher orients the student to TAPES. The student is taught to recognize the three signs of anxiety and practices identifying his or her own signs with the teacher and parent. Next, the CBT model of anxiety reduction is reviewed and the student is taught to use a journal to identify and record his or her own signs of anxiety. Finally, the teacher introduces relaxation skills to target the physical symptoms of anxiety and practices deep breathing and progressive muscle relaxation with the parent and student.

##### Meeting 3: facing fears

The teacher reviews the three signs of anxiety and provides a rationale for exposure or facing fears (referred to as “brave” behaviors). The group completes a personalized list of brave behaviors based on the student’s avoidance at home and school, which provides the basis for school and home bravery charts. These charts function as daily report cards to track the student’s progress on behavioral exposures. Rewards are assigned for engaging in brave behaviors as needed.

##### Meeting 4: coping thoughts

In meeting four, the teacher checks in regarding the school and home bravery charts and problem solves any difficulties that have occurred (e.g. student avoided the brave behaviors, rewards were not motivating enough). Next, the teacher introduces the skill of identifying cognitive distortions and teaches the student and parent methods for challenging these maladaptive thoughts and replacing them with “coping” thoughts in various anxiety-provoking situations. Lastly, new brave behaviors are chosen for the upcoming weeks.

##### Meeting 5: planning ahead and coping with future anxiety

In the final TAPES meeting, the teacher reviews the bravery charts and highlights the student’s progress in the program (e.g. skills learned and successes to date). The content of this meeting focuses on future situations when anxiety may become excessive and developing a coping plan to help prepare the parents and student for anticipated challenges. The student, parent, and teacher collaboratively develop a comprehensive coping plan that utilizes the skills learned in TAPES.

##### Classroom component

In addition to the school–home meetings, teachers are taught anxiety-reduction strategies that can be applied classroom-wide to improve classroom climate. Teachers are given sample scripts and additional resources (i.e. websites, links to videos) that can be used proactively, to strengthen students’ existing coping skills, and responsively, to address anxious behaviors exhibited by students. As emotionally supportive environments have been found to be a protective factor for anxious students [30], teachers are also given strategies to increase prosocial interactions amongst students, encourage teamwork and collaboration, and ensure that positive behavioral strategies are used consistently.

##### Relationship component

Research on the role of teacher behavior in the development and maintenance of child anxiety suggests that teachers who exhibit highly controlling behaviors, such as issuing frequent directives, tend to increase child anxiety and negatively affect children’s ability to learn [31]. Therefore, teachers are taught to recognize behaviors that can either promote or reduce student anxiety. Along with the parents in the school–home meetings, teachers are challenged to evaluate and modify their own behavior with the anxious student. Lastly, because positive teacher–student relationships have found to protect youth from developing internalizing behavior problems over time [32], teachers are given strategies to improve the quality of their relationship with the student and the student’s parents, which are centered around core interpersonal skills (Respect, Empathy, Listening, Acknowledging efforts, Teamwork, and Encouragement or RELATE).

#### Theory of teacher behavior change

**Fig. 1:**
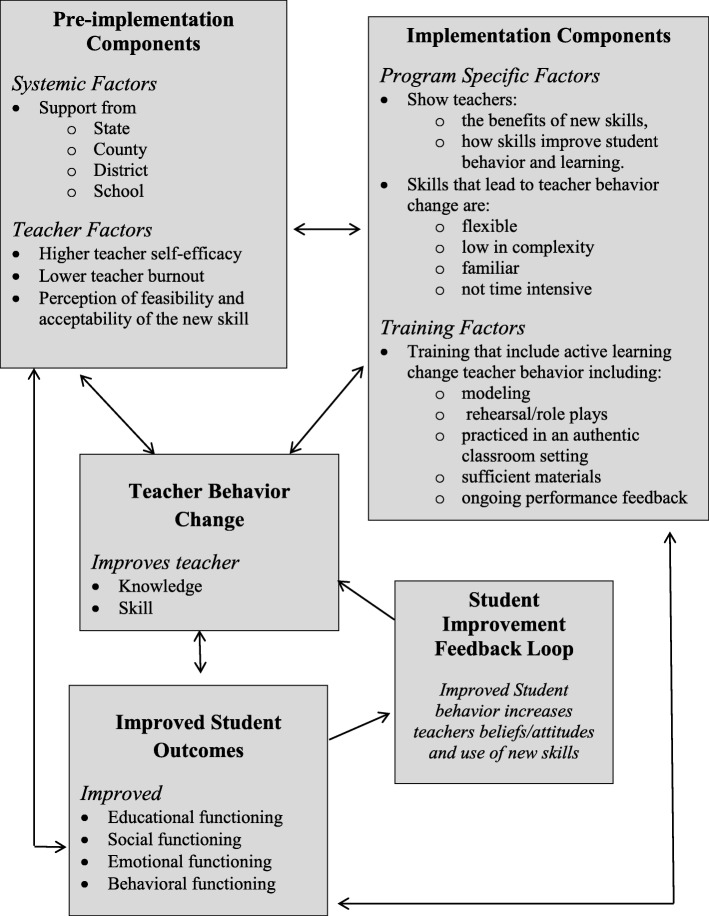
Theory of Change for TAPES

**Fig. 2:**
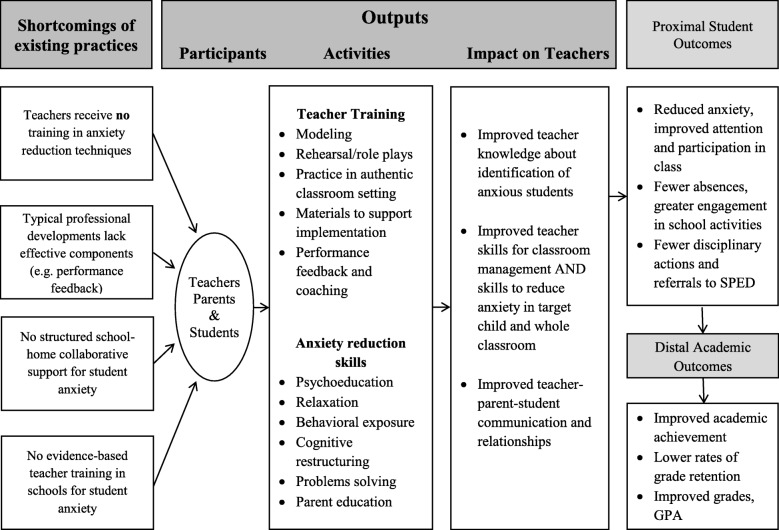
Logic model for TAPES

The proposed theory of change for TAPES and logic model (see Figs. 1 and 2) were guided by extant research and models proposed by Han and Weiss [33] and Guskey [34], who detail mechanisms of teacher behavior change in adopting mental health interventions. The components of these models focus on pre-implementation (e.g. state, county, district, and school priorities and policies) and implementation factors (e.g. ongoing performance feedback) that have been associated with teacher behavior change. In addition to these systemic issues, several teacher factors influence teacher adoption, implementation, and sustained use of new skills [35]. These include higher teaching self-efficacy (i.e. teachers’ beliefs that they are capable of implementing the new skills; [36]) and teacher burnout, a factor which negatively affects teachers’ attitudes toward and interactions with students and increases indifference and hostility [37]. Both will be measured and addressed in TAPES. Finally, teachers’ perceptions of the feasibility and acceptability of the new skills [38] impact behavior change. Specifically, teachers’ understanding of and beliefs that the new skills will solve an important student problem, are efficacious and are compatible with their teaching style and beliefs about children’s behavior [39] all increase behavior change [40, 41].

#### Theory of change related to student outcomes

Etiological models of anxiety propose that these disorders involve excessive physiological arousal, cognitive distortions, and behavioral components such as avoidance of feared stimuli [29]. Thus, the underlying theory of TAPES’ CBT strategies is that teacher-facilitated change in hyperarousal, maladaptive cognitions, and avoidant behavior in the classroom, will result in the reduction of anxiety and improvement in academic outcomes. The mechanisms by which anxiety exerts a negative impact on academic performance are poorly understood and have rarely been studied. Some propose that higher levels of anxiety increase physiological arousal and shift the focus of attention away from classroom instruction and toward threat cues in the environment, thus impairing concentration and working memory, and ultimately undermining children’s ability to recall previously learned material [42, 43]. Indeed, findings from one study suggest that anxiety negatively impacts learning by interfering with working memory [43]. Data supporting the theoretical model of the CBT components used in TAPES come from a large treatment literature of childhood anxiety disorders (see [44] for a review). It is hypothesized that through modifying teacher behavior, enhancing the generalization skills across school and home (through the conjoint meetings), and fostering improved communication between teachers and parents as they work on shared goals will result in positive child outcomes [45].

#### TAPES training and consultation

Teachers randomized to TAPES will participate in a one-day in-person training (approximately 6 h), which includes active/experiential learning strategies, opportunities for observation (via video clips), live modeling and role plays, and coached practice. Teachers will be offered 30-min of weekly consultation by the study team to improve intervention fidelity and quality. Consultation will include case review, skill rehearsal, problem-solving obstacles, and feedback regarding performance based on audiotaped sessions.

#### Comparison condition: Teacher Anxiety Training (TAT)

The TAT is a 3-h didactic training on student anxiety adapted from presentations used in the PIs’ ongoing school-based studies for school clinicians and nurses. The TAT content will include information on the signs, causes, consequences, and effective interventions for student anxiety. This comparison condition was selected to enhance teacher recruitment and mimic the format of typical teacher professional development trainings in CT. Thus, we anticipate that TAT will provide a credible and acceptable control. The impact of TAT on teacher behavior and child outcomes relative to TAPES will be examined in the RCT.

### Measures

The selection of proposed measures was guided by the best available measures for the study aims and proposed theory of change. Data will be collected from multiple informants (i.e. teacher, independent evaluator, parent, and student) using multiple formats (e.g. classroom observations, rating scales, interviews, school records). Measures, informants, and timepoints are listed in Table 1. No biological samples have or will be collected in this trial or any future studies related to this trial.

**Table 1:** List of study measures

Instrument	Rater	Timepoint	Condition
Teacher knowledge and skills			
Teacher Knowledge Assessment Form A and Form B	Teacher	BL, PO	TAPES & TAT
Classroom Observation of Teacher Skills	IE	BL, PO, FU	TAPES & TAT
Fidelity of implementation measures			
School-Home Fidelity and Quality Measure (Meetings 1–5)	Study staff	WKLY	TAPES
Teacher S-H Meeting Summary Form	Teacher	WKLY	TAPES
Teacher and student measures linked with theory of change			
Student-Teacher Relationship Scale	Student, Teacher	BL, PO, FU	TAPES & TAT
Parent-Teacher Relationship Scale	Parent, Teacher	BL, PO, FU	TAPES & TAT
Teacher Efficacy Beliefs Scale	Teacher	BL, PO, FU	TAPES & TAT
Teacher as Social Context (TASC)	Student	BL, PO, FU	TAPES & TAT
Teacher Background Form	Teacher	BL	TAPES & TAT
Maslach Burnout Inventory – Educators Scale (MBI-ES)	Teacher	BL, PO, FU	TAPES & TAT
Organizational Readiness Questionnaire	Teacher	BL	TAPES & TAT
Woodcock-Johnson IV Numbers Reversed Subtest	IE	BL, PO, FU	TAPES & TAT
Family Accommodation Scale, Anxiety (FASA)	Parent	BL, PO, FU	TAPES & TAT
Teacher Accommodation Scale, Anxiety (TASA)	Teacher	BL, PO, FU	TAPES & TAT
Student educational achievement			
School Records Form	Study staff	BL, PO, FU	TAPES & TAT
Woodcock-Johnson IV Tests of Achievement (WJ IV)	IE	BL, PO, FU	TAPES & TAT
Student school and classroom behavior			
Student Attendance and Services Form	Teacher	WKLY	TAPES & TAT
School Anxiety Scale, Teacher Report (SAS-TR)	Teacher	BL, PO, FU	TAPES & TAT
School Connectedness	Student	BL, PO, FU	TAPES & TAT
School Refusal Questionnaire	IE	BL, PO, FU	TAPES & TAT
Student mental health			
Anxiety Disorders Interview Schedule for DSM-5 Parent and Child Versions (ADIS-5-C)	IE	BL, PO, FU	TAPES & TAT
Clinical Global Impression – Severity (CGI-S) Scale	IE	BL, PO, FU	TAPES & TAT
Clinical Global Impression – Improvement (CGI-I) Scale	IE	PO, FU	TAPES & TAT
Children’s Global Assessment Scale (CGAS)	IE	BL, PO, FU	TAPES & TAT
Spence Children’s Anxiety Scale, Child and Parent Versions (SCAS)	Student, Parent	BL, PO, FU	TAPES & TAT
Strength and Difficulties Questionnaire-Teacher version (SDQ)	Teacher	BL, PO, FU	TAPES & TAT
Teacher Observation of Classroom Adaptation-Checklist, Concentration Problems scale (TOCA-C)	Teacher	BL, PO, FU	TAPES & TAT
Avoidance Hierarchy	IE, Parent, Teacher	BL, PO, FU	TAPES & TAT
Additional study measures			
Demographics Form	Parent	BL	TAPES & TAT
Service Utilization Form	IE	BL, PO, FU	TAPES & TAT
Patient Health Questionnaire (PHQ)	Parent, Teacher	BL, PO, FU	TAPES & TAT
School Attendance, Discipline, and Parent’s Missed Work	Parent	BL, PO, FU	TAPES & TAT

### Teacher knowledge and skills

- Teacher Knowledge Assessment Form A and Form B is a 25-item short answer and multiple-choice assessment of anxiety reduction strategies to gauge teachers’ knowledge and the effectiveness of training. This measure was adapted from an existing knowledge assessment for school clinicians, which showed an increase in knowledge from pre- to post-training [46]. Form A will be completed by teachers before the TAPES and TAT trainings. Form B will be administered at the post timepoint to measure teachers’ retention of knowledge and skills following the eight-week intervention period. After calculating the percentage of points correct out of points possible, aggregate means and standard deviations (SD) will be used to examine change in teacher knowledge from pre- to post-intervention.
- Classroom Observation of Teachers Skills is a form completed during direct observations of the teacher during normal class activities (e.g. math or reading). Classroom observations will be conducted to evaluate change across three timepoints: (1) before the TAPES and TAT trainings (baseline); (2) after the administration of TAPES and TAT (eight weeks); and (3) at the follow-up assessment. Across each 30-min observation period, independent evaluators tally the frequency of specific behaviors that are theorized to increase student anxiety (e.g. hostility, over-control), as well as specific behaviors that are theorized to decrease anxiety (e.g. warmth, autonomy promotion). Using a 5-point Likert scale, observers provide an overall rating of the teacher’s behavior in both these domains. Means and SDs will be calculated for each domain and compared across timepoints.

### Fidelity of implementation measures (TAPES teachers only)

- School-Home (S-H) Fidelity and Quality Measure (Meetings 1–5) is a form completed by a study member (generally within 48 h) after each meeting to assess the fidelity and quality of TAPES skills. Meetings will be rated after listening to audio-recordings sent by the teacher to the study team. The goals of each meeting are rated for adherence (Was goal accomplished? Yes/No) as well as quality of implementation (1 = poor to 4 = very good), which reflects the accuracy of the presentation, use of elaboration and student-specific examples, and assessment of parent/student understanding. Adherence for each meeting will be measured in percentage of goals accomplished out of total number of goals for that meeting (e.g. completing 4 of 5 meeting goals equals 80% adherence). All meetings will be coded for quality of implementation and will yield an overall mean score and SD.
- Teacher S-H Meeting Summary Form is a 16-item measure completed by the teacher following each school–home meeting. This checklist contains items that measures parent and student attendance, involvement, assesses barriers to use, and whether goals were accomplished. Using a 7-point Likert scale, teachers will provide a rating of parent and student involvement, teacher confidence, and overall student compliance. Means and SDs will be analyzed quanitatively to provide information about the feasibility of the TAPES intervention.

### Teacher and child measures linked with TAPES theory of change

Several measures will be collected to assess factors that may influence fidelity of TAPES skills and/or are directly linked to the proposed theory of change:

- The Student-Teacher Relationship Scale [47] is a 15-item scale with good psychometric properties that will assess the quality and change in the teacher–student relationship across baseline, post, and follow-up. This measure is completed by teachers and will be used to generate mean and SDs that can be compared across timepoints.
- The Parent–Teacher Relationship Scale [48] is a 24-item measure of teacher–parent relationship quality and will be used to measure the change in relationship quality across baseline, post, and follow-up. The measure will be completed by parents and teachers, independently, and will generate mean and SD scores that can be compared across time. This measure has good psychometric properties [49].
- The Teacher Efficacy Beliefs Scale [35] is a 12-item psychometrically sound scale of teacher efficacy such as efficacy for classroom management and student engagement. It will be completed by teachers at baseline, post, and follow-up. Means and SDs will be calculated based on sum of all items in order to examine change in teacher efficacy across time [50].
- The Teacher as Social Context (TASC) [51] teacher involvement scale and help/support subscale will be used to assess the student’s perception of his or her teacher’s affection, attunement, dedication, and dependability. Using a 4-point Likert scale, the student will provide a rating of their teachers’ improvement and help/support. The student will complete this measure at baseline, post, and follow-up; means and SDs will be utilized to assess change.
- The Teacher Background Form is a 16-item measure completed at baseline that assesses teachers’ demographic characteristics (e.g. age, gender, race) and professional experience (e.g. degree, training, years teaching, class size, and confidence in reducing anxiety). These data will be used to describe the sample of participants in the RCT.
- The Maslach Burnout Inventory – Educators Scale (MBI-ES) [37] is a 22-item measure with strong psychometric properties that assesses teacher burnout [52]. This measure utilizes a 6-point Likert scale and is completed by all teachers at baseline, post, and follow-up. Mean and SDs will be calculated to assess change across time.
- The Organizational Readiness Questionnaire will be used to assess teacher perceptions of the overall climate of his or her school at baseline. This 25-item measure was adapted from the organizational climate scale of the Texas Christian University Organizational Readiness for Change measure [53]. Teachers will provide answers using a 5-point Likert scale. Total mean scores will be evaluated as a potential moderator of teacher and student outcomes.
- The Woodcock-Johnson IV Numbers Reversed Subtest [54] will be used to assess verbal working memory at baseline, post, and follow-up. This measure is included to explore the relationship between anxiety and working memory [17, 42, 43], which has been hypothesized to account for academic impairment among anxious youth. Performance on this subtest is reported as a standard score. Means and SDs will be examined for changes across timepoints.
- The Family Accommodation Scale, Anxiety (FASA) [55] is a 13-item measure that provides ratings of parent’s participation in anxiety-related accommodation behaviors, modification of family functioning, and related family distress. The FASA is completed by parents or caregivers, and it has good internal consistency and demonstrates convergent and divergent validity [55]. This measure utilizes a 5-point Likert scale to assess level of accommodation and distress. Parents will complete this measure at baseline, post, and follow-up, and total means and SDs will be used to assess change over time.
- The Teacher Accommodation Scale, Anxiety (TASA) is a modified version of the FASA developed for this study. The TASA will be administered to teachers at baseline, post, and follow-up evaluations to obtain frequency ratings of teacher accommodation behaviors in the classroom. Teachers will provide ratings of anxiety-related behaviors in the classroom, modification of classroom routines and/or individual responsibilities, and related distress. Similar to the FASA, this measure utilizes a 5-point Likert scale and will be used to generate a total mean and SD score for comparison across all timepoints.

### Student educational achievement

- The School Records Form will be used to assess information on grades and attendance. Obtaining records directly from the student’s school, the study team will record the student’s absences, tardy arrivals, and early dismissals. In addition, grades or academic marks will be converted to a 4-point Likert scale for comparison across grading systems. School records will be collected and coded at baseline, post, and follow-up. Using these data, means and SDs will be calculated to assess change across timepoints.
- The Woodcock-Johnson IV Tests of Achievement (WJ IV) [56] is a widely used, norm-referenced measure of academic achievement. Several subscales (reading, writing, and math fluency) are believed to be affected by anxiety. The measure has strong psychometric properties [57]. Students will be administered the academic fluency subtests at baseline, post, and follow-up. Standard scores will be calculated for all three subtests and changes in these scores will be assessed over time.

### Student school and classroom behavior

- The Student Attendance and Services Form will be used to assess weekly student attendance during the intervention period as well as referrals and utilization of additional academic (e.g. IEPs, Section 504 plans) and mental health services. Teachers complete this form for eight consecutive weeks after baseline. After totaling the number of services utilized during the intervention period, group comparisons will be used to compare differences between students receiving either TAPES or TAT.
- The School Anxiety Scale, Teacher Report (SAS-TR) [58] is a 16-item questionnaire that assesses anxiety related behaviors in the classroom (e.g. child is afraid of asking questions). The SAS-TR has acceptable psychometric properties (e.g. alpha for total score was 0.93). The teacher will complete this questionnaire at baseline, post, and follow-up. Total scores will be examined to assess change across timepoints.
- School Connectedness [59] is a widely used five-item questionnaire completed by the student asking about their feeling towards school [60]. The student will complete this measure at all timepoints (baseline, post, follow-up). Using this measure, overall mean and SDs will be calculated and group comparisons will be made at each timepoint.
- The School Refusal Questionnaire is a 12-item measure adapted from the Anxiety Disorders Interview Schedule for DSM-5, Child Version (ADIS-5-C) [22] that provides information about school absences, early dismissals, and school nurse or counselor visits attributed to anxiety. These items will be administered during the parent interview at the baseline, post, and follow-up assessments. Items are scored dichotomously (yes/no) and on a Likert-scale capturing frequency. Mean values will be compared between groups at each timepoint.

### Student mental health

- Anxiety Disorders Interview Schedule for DSM-5 Parent and Child Versions (ADIS-5-C) [22] is considered the gold standard for assessing anxiety diagnoses and severity. Impairment ratings are generated for each disorder using the Clinician Severity Rating (CSR; range = 0–8; ≥ 4 required to assign a diagnosis). The previous version of this assessment, the ADIS-IV-C, has good test–retest reliability for the parent interview (*r* = 0.98) and for the child interview (*r* = 0.93) [61] and is sensitive to intervention effects [62, 63]. The parent and child interview will be administered at baseline, post, and follow-up. Information provided in the interview will be used to inform diagnostic status assigned by the independent evaluator. Changes in CSR scores and diagnostic status will be compared between groups across timepoints.
- Clinical Global Impression – Severity (CGI-S) and Improvement (CGI-I) Scales [64]. The CGI-S is a measure of global anxiety severity ranging from 1 (normal, not at all ill) to 7 (extremely ill). A rating is provided by the independent evaluator and will be compared between groups across timepoints (baseline, post, follow-up). The CGI-I is assigned at post and follow-up assessments only to provide a global rating of clinical improvement in anxiety symptoms since the baseline assessment. Scores on the CGI-I range from 1 (very much improved) to 7 (very much worse). Both measures are widely used in child treatment trials to assess symptom severity and improvement [65]. Mean group scores will be used to assess change across groups over time.
- The Children’s Global Assessment Scale (CGAS) [66] is used to describe a child’s global impairment and functioning at home, school, and with peers on a scale of 1 (gross impairment) to 100 (superior functioning). The CGAS has been used in child anxiety treatment studies to monitor changes in global functioning [67]. A CGAS rating will be assigned by the independent evaluator at baseline, post, and follow-up. Mean group scores will be used to compare changes in overall functioning at each timepoint across groups.
- Spence Children’s Anxiety Scale, Child and Parent Versions (SCAS) [21, 68] is a 38-item measure rated for frequency of occurrence (0 = never to 3 = always) of a broad range of anxiety symptoms. The SCAS–C and SCAS–P have sound psychometric properties, with internal consistency reported at 0.89 for the total parent anxiety score and 0.92 for the total child score [69–71]. This measure was selected to facilitate comparisons with published school-based studies using the FRIENDS program [10, 11, 72]. Parents and students will each complete the SCAS at baseline, post, and follow-up. The scale uses a 4-point Likert scale and yields a total summed score. Total score means and SDs will be used to compare changes in anxiety symptoms across time.
- The Strength and Difficulties Questionnaire- Teacher version (SDQ) [73] is a 25-item widely used questionnaire about children’s classroom behavior. The teacher-report version has sound psychometric properties [74]. The teacher will complete this measure at baseline, post, and follow-up. Items are rated on a 3-point Likert scale. Total mean scores will be used to compare students’ behavior across all timepoints.
- The Teacher Observation of Classroom Adaptation-Checklist, Concentration Problems scale (TOCA-C) [75, 76] is a measure of student behavioral adjustment that demonstrates high internal consistency and construct validity. The seven-item Concentration Problems scale will be used to assess inattentive behaviors in the classroom. Teachers will complete this measure at all timepoints and total mean scores will be used to evaluate change over time across groups.
- Avoidance Hierarchy is a measure of the student’s top three most frequently avoided situations at home and at school, which are assigned and rated by the independent evaluator at baseline. Each behavior is rated on a 7-point Likert scale to assess how often the student avoids engaging in the behavior (1 = never avoid to 7 = avoid every time). The independent evaluator rates the same items created at baseline again at post and follow-up assessment. This numeric data will be used to assess change in behavioral avoidance at post and follow-up compared to baseline.

### Additional study measures

- The Demographics Form is a questionnaire assessing child and family information such as child age, family income, parental education, race/ethnicity, and other characteristics. This information will be collected at baseline and will be used to describe the participants in the sample.
- The Service Utilization Form is a questionnaire administered by the independent evaluator at each assessment to document involvement or changes in psychological and/or psychiatric services. Services are coded by frequency and type. This questionnaire is administered at all timepoints in order to assess change in the use of total number of services at post and follow-up relative to baseline.
- The Patient Health Questionnaire (PHQ) is a 15-item questionnaire that will be used to assess parent and teacher anxiety and mood symptoms. This form was adapted from two screening measures (GAD-7 and PHQ-9) that have shown good reliability and validity in primary care settings [77, 78]. Parents and teachers will each complete this form at baseline, post, and follow-up. Total mean scores will be calculated and may be used to compare anxiety and mood symptoms over time; baseline scores may also be examined as a moderator.
- School Attendance, Discipline, and Parent’s Missed Work is a questionnaire that obtains information from parents about their child’s school attendance and disciplinary record, as well as the amount of work that parents have missed due to the child’s anxiety. Parents will complete this questionnaire at baseline, post, and follow-up. Numeric data will be used to calculate total mean scores that will be evaluated for change over time.

### Independent evaluator (IE) training

IEs will have a masters or doctoral degree in a relevant child mental health field and experience with conducting diagnostic assessments with anxious youth. Training and certification of IEs will include completion of: (1) didactic training that includes review and practice of all assessment measures and study procedures by the PIs; (2) review of two ADIS-5-C videotaped administrations by a senior interviewer; (3) achieving inter-rater reliability (kappa) of 0.85 for primary diagnoses and severity ratings on three cases (live or with videotapes); and (4) administration of the ADIS-5-C in the presence of a senior interviewer. All assessments will be videotaped and a random 15% of tapes will be evaluated for interrater reliability (i.e. for the ADIS-5-C diagnoses and severity ratings).

### Procedures

Recruitment efforts will utilize flyers and communication with school administrators and personnel. Specifically, the recruitment process will involve the following: (1) the study team will contact public school districts starting from within a 20-mile radius from the University of Connecticut, USA; (2) school districts that agree to participate will email teachers an IRB-approved study flyer or place paper flyers in teachers’ mail boxes. Depending on teacher response (40 teachers are needed), additional districts that are further from the university will be approached. Teachers will be consented and enrolled on a rolling basis. If > 40 teachers express interest, the research team will randomly select teachers from the pool of interested teachers until the recruitment goal of 40 is met.

**Fig. 3:**
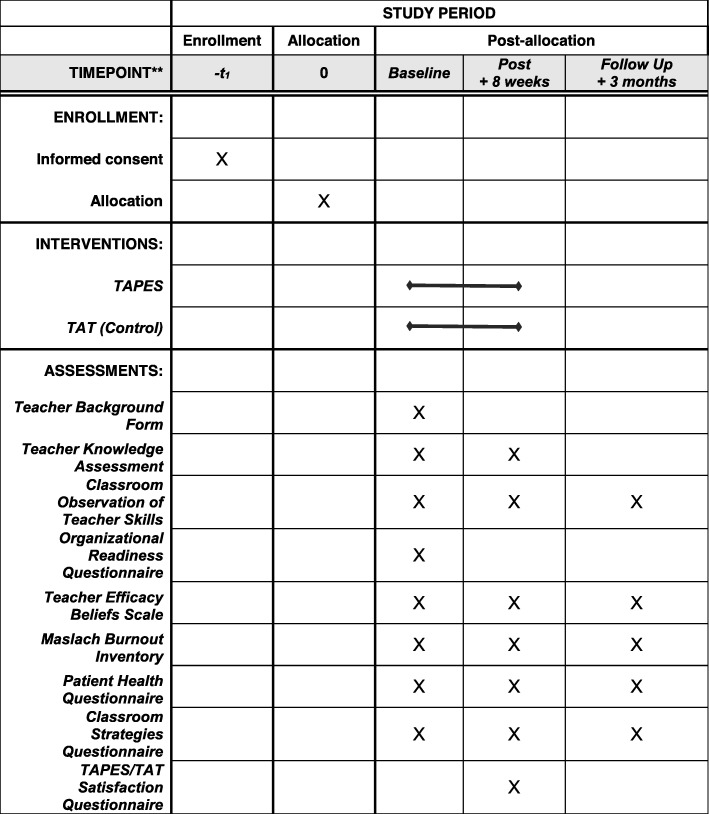
Table of teacher timeline for enrollment, interventions, and assessments of primary outcomes

Teachers will be randomly selected from the pool of interested participants and randomized (1:1 allocation) to TAPES or TAT (see Fig. 3 for teacher timeline and Additional file 1 for the Standard Protocol Items: Recommendations for Interventional Trials [SPIRIT] checklist). Once teachers have completed baseline questionnaires and been randomized by the study coordinator, teachers will complete their assigned training. Teachers will then identify potentially eligible students from their classes and provide information about the study to their parents. Interested parents will contact study staff and complete a brief phone screen. Students who appear eligible based on the phone screen (e.g. have elevated anxiety; are in elementary school) will be invited to complete informed consent (and child assent) and a full baseline evaluation with an IE at the school or research office (see Table 1 for measures). Families who “pass” the baseline evaluation (i.e. student obtains a *t* score of ≥ 60 on the SCAS and/or a severity rating on the Anxiety Disorders Interview Schedule of ≥ 3) will be considered eligible. Depending on their random assignment, teachers will implement either TAPES or TAT over eight weeks with the enrolled student.

After eight weeks, teachers, parents, and students in both groups will complete a post evaluation completed with an IE who is blind to condition (see Table 1 for Measures). A three-month follow-up evaluation to assess sustained use and impact of TAPES/TAT skills will also be conducted with an IE blind to study condition.

In order to promote retention and study completion and compensate for time away from daily tasks, participants will receive reimbursements. Teachers will be reimbursed for attending their training ($50) and will receive $25 for completing the baseline, post, and follow-up measures on each student (total of $75 per student). Families will receive $20 gift cards after completing the baseline evaluation, $40 for the post evaluation, and $40 for the follow-up evaluation (total of $100 per family). Efforts will be made to collect data at each assessment from all participants regardless of whether they discontinued receiving the interventions.

Implementation of the trial will be monitored by the study team during weekly meetings that will assess recruitment progress, data quality, and ensure appropriate training of new staff. The study team comprises the PIs, data manager, study coordinator, and research assistants (RAs). Audits of the trial by an external data monitoring committee are not planned due to the minimal risks involved in this trial but may be conducted at random by the university’s institutional review board and/or funder. Adverse events are collected at each evaluation by the independent evaluator. Any identified events are reported to the PI, university IRB, and funder. Criteria for discontinuing the study include participant request and/or non-compliance with study procedures (e.g. refusal to attend meetings). Students who display a worsening of symptoms will be referred for mental health services (though, as per consent form, no compensation is awarded to those who suffer harm from participation). Students who need post-trial care will be referred for clinical services in their school and community by the research team when they conduct their final assessment (or prior if needed). At the end of their participation in the trial, the majority of students will be with a new teacher who may or may not be participating in the study. Teachers trained in the study are free to use their new skills with all students in their classroom. Teachers trained in TAT—the comparison condition—will not receive additional training.

Assigned condition will remain masked to IEs during the trial. Modifications to the protocol will be tracked by the study team during weekly meetings and reported to the IRB on an ongoing basis. Major modifications will also be communicated to participants, clinicaltrials.gov, and reported in journals upon publication of findings.

#### Randomization

The study design is a parallel RCT. Randomization will occur at the teacher level at a 1:1 (20 TAPES: 20 TAT) following consent. The randomization plan will be generated by the study team using a pre-populated web-based randomization plan generator before the identification of teachers to minimize potential biases. Teachers will be matched on grade level before randomization and allocation will be conducted by the study team. The sequence of randomization is numbered sequentially but is not concealed to the research team. Randomizing teachers to two active training conditions with parallel group implementation was selected for several reasons. The current design allows for all teachers to receive some training in anxiety identification and reduction to account for a major recruitment barrier in our previous school-based trials where clinicians or nurses were assigned to a no-training control condition and subsequently dropped from the study. In addition, this design allows for an examination of a brief teacher training which represents typical teacher professional development offerings in CT. We recognize there is a risk of intervention contamination randomizing teachers within the same school and plan to carefully monitor this at each assessment by asking teachers, parents, and students what interventions they received since their last evaluation. In addition, teachers will be asked to refrain from sharing intervention materials with colleagues in their school until the end of the study in order to minimize the threat of contamination. Finally, given the intensity and structure of TAPES (i.e. five individual meetings with parent and child), which varies significantly from usual teacher practices, we considered it unlikely that teachers would implement this component in the comparison group.

#### Data management

Each participant will be assigned a unique identification (ID) number. This ID number, rather than names, will be used on all files and forms to protect confidentiality. At the end of every evaluation, an RA will check all forms and databases to assess for completeness and quality. If necessary, the data will be corrected for errors (e.g. missing items) and entered into a database. Weekly meetings will be held to alert staff of any issues identified during the checking process and to review and correct all errors. All data will be password protected and backed up monthly. At each stage of data collection and maintenance, measures will be taken to ensure that all identifying information is omitted from data archives, that hard copies of data are stored in locked file cabinets with restricted access, and that computer files are password-protected. Copies of all data files will be maintained on external drives. Redundant copies of the drives will be stored in a separate off-site cabinet to ensure the survival of the data in case of fire or other disaster. Backups will be made monthly. During the study, access to data will be restricted to the research team.

#### Data analytic plan

Preliminary analyses will include: (1) evaluating the psychometric properties of measures; (2) verifying assumptions in analysis of covariance (ANCOVA), general linear model (GLM), and generalized linear mixed model (GLMM); and (3) examining the nature of missing data (e.g. MAR). Specifically, we will run descriptive analyses to check for outliers to ensure that distributional assumptions of the planned analyses are appropriate. If not, analogous non-parametric methods will be used. Although all of our primary measures have a favorable psychometric history, we will ensure a satisfactory level of internal consistency for each measure through calculation of Cronbach’s alpha. With respect to missing data, we will: (1) make every possible effort to prevent missing data; (2) use the intention-to-treat (ITT) principle and all available data in analyses; and (3) conduct sensitivity analyses that assess the robustness of the results. IEs and RAs will review all assessment materials in the presence of teachers, children, and parents to limit the amount of missing data and to help verify the correctness of the data. Because missing data may lead to biased estimation and loss of statistical power if handled inappropriately, we will draw upon several approaches if missing data are present. For instance, we will conduct a partial check to determine if the data are MAR by evaluating whether “missingness” can be explained on the basis of measured variables. Multiple imputation (MI) has been shown to improve upon traditional simple methods for handling missing data (e.g. list-wise deletion, mean substitution). Thus, MI ITT results will be compared with other methods such as complete case or maximum likelihood methods in a sensitivity analysis [79].

To assess the impact of TAPES, compared to TAT, on teachers’ knowledge and skills and student outcomes, we will first assess the equivalence of TAPES and TAT on baseline teacher (years of experience, burnout, teacher efficacy, teacher behavior) and child characteristics (e.g. gender, age, baseline anxiety symptoms, classroom functioning). Next, we will assess adherence of the intervention and any contamination between them based on TAPES fidelity measures and post evaluations from student, parent, and teacher in both TAPES and TAT conditions. To compare the effectiveness between TAPES and TAT, teacher and student outcomes at baseline, post-intervention, and three-month follow-up will be modeled using GLMM. Teacher-specific (e.g. years of teaching experience, teaching efficacy) and child-specific (e.g. age, baseline anxiety severity) covariates will be added as needed. To examine mediators (proposed in the theory of change, see Fig. 1), predictors and moderators structural equation modeling (SEM) will be used. No interim analyses are planned. Additional analyses (e.g. examining subgroups) are not currently planned and thus will be exploratory. Access to the final de-identified trial dataset will be available to the study statistician and funder.

#### Power analysis

Using Optimal Design version 3.0, calculations were based on an anticipated enrollment of 20 teachers in each group (and 1–2 students per teacher). We assumed a conservative within-cluster intraclass correlation coefficient of 0.10 [80] and an alpha level was set at 0.05. With 20 teachers in each group, we will have 80% power to detect the effect size found by White et al. [81] of *d =* 1.19 on teacher knowledge. With 30 students in each group, we will have 80% power to detect the effect size found by Barret et al. [10] of *d =* 0.92 on child anxiety (effect sizes based on moderately anxious students at pre-post time points).

## Discussion

Excessive student anxiety is a common problem that severely impairs short- and long-term academic functioning and increases teacher burden. Teachers lack training in anxiety-reduction skills needed to support the learning of students with excessive anxiety. Reducing student anxiety has been associated with improvement in educational functioning. Because anxiety manifests daily in the classroom, teachers are in an ideal position to identify and help students manage their anxiety. The aim of this protocol is to address this gap by assessing the feasibility and impact of a novel teacher-delivered school–home intervention to assist anxious students. The impact of TAPES will be compared to a standard professional development condition on teacher and student outcomes. Exploratory aims will examine potential mediators. If effective, TAPES has the potential to directly benefit: (1) teachers—by providing training in an important and relevant, but neglected area that will enhance their professional development and effectiveness in the classroom; and (2) students—by reducing their anxiety and improving their educational, social, and behavioral functioning.

In light of the high prevalence of child anxiety and associated academic deficits, the majority of anxious students need specialized educational and mental health interventions [82]. However, more youth need services than receive them [83] and access to evidenced-based services is poor. Enhancing teachers’ capacity to support these students can potentially reduce this service gap and improve academic outcomes.

The consequences of the existing gap in teacher knowledge and skills to assist anxious students are significant for both teachers and students. Teachers with minimal training in identifying and managing students’ anxiety are less likely to be effective in: (1) providing appropriate accommodations and modifications to support the anxious student’s ability to succeed academically and socially; (2) minimizing disruptions to instruction and other students’ learning; and (3) maintaining a positive teacher–student relationship, as teachers may struggle with methods to engage and motivate these students [84]. Conversely, when teachers are equipped with appropriate knowledge and skills, they have a significant and positive effect on students’ social-emotional, behavioral, and educational functioning both concurrently and prospectively [85–88]. Moreover, when teachers experience mastery over students’ social and emotional challenges, such as excessive anxiety, teaching becomes more enjoyable, teachers feel more efficacious and there are improvements in teacher–student relationship quality [89, 90].

Although TAPES has the potential to help teachers, students, and families, there are some limitations to the program. First, although the program includes a classroom-wide approach to anxiety reduction, the program is primarily focused on the school–home collaborative approach and is principally designed to be used with one student at a time. Given the prevalence of clinical and sub-clinical levels of anxiety, the demand for services might exceed the level of support offered in TAPES. However, if the program is successful at increasing teacher knowledge and skill, teachers will be able to generalize and apply these skills to multiple students within their classrooms or to use with small groups during the school day.

Second, there is the possibility that students participating in TAPES may be currently receiving additional services during the intervention period. In an effort to have a more inclusive and representative sample, students are not excluded if they are receiving outside services. While randomization at teacher level may address this potential confound, student outcomes could be contaminated by additional services, such as pharmacological treatment or outpatient CBT. These will be measured and controlled in analyses.

With regards to teacher outcomes, the sample of teachers that volunteer to participate in TAPES may not be representative of the teacher population as a whole, as volunteers are willing to devote additional time beyond their normal work duties to train on this program raising concerns about the generalizability of findings to the larger population of teachers. Moreover, as TAPES is a novel intervention, there is a lack of established measures designed to evaluate teacher knowledge and behavior change specifically linked to anxiety reduction strategies. Finally, although both teacher (observed teacher skills in the classroom) and student outcomes (anxiety severity) will be assessed using blinded/masked evaluators, additional outcomes will be reported by study participants (teachers, parents, youth). The central limitation of unblinded assessment of outcomes is that there may be potential bias by these reporters who know they are receiving an active intervention. The current study minimizes this bias by including two credible intervention conditions. In addition, the data analyst / statistician will be blind to intervention condition. RAs will also be blinded to intervention condition (PIs and study coordinator will not be blind to study condition). Interpretations of findings assessed by unblinded informants will be done cautiously.

The current TAPES model is based on research demonstrating that successful teacher trainings include active learning approaches, such as modeling and rehearsal/role plays, user-friendly materials to support implementation (e.g. manuals, handouts), and ongoing performance feedback. Within the current study, teachers receive support from study staff in identifying and evaluating anxious students, personalizing intervention materials to address the student’s fears and worries, and troubleshooting difficulties with implementation. To ensure the sustainability of TAPES, future research is needed to evaluate the ability of school personnel (e.g. school psychologists, administrators) to serve as trainers and coaches.

In conclusion, the current paper proposes a novel teacher-led school–home intervention to help reduce student anxiety in the classroom and at home. If effective, this program could facilitate the dissemination of cognitive behavioral techniques to a new provider, thereby expanding student access to evidenced-based intervention. Toward this end, findings from the current study will be disseminated via publications in relevant professional journals, at national and international conferences, and to local school boards and study participants. The results of this project have potentially important benefits for teachers and provide a new wave of defense against the deleterious academic, social, and emotional consequences of pediatric anxiety.

## Trial status

ClinicalTrials.gov, NCT03899948.

Last date of approval by UCONN IRB 09/09/19; Protocol Version 19.

Recruitment began: 5/8/2018; approximate recruitment end date: 1/31/2021.

## Supplementary information

**Additional file 1.** SPIRIT 2013 Checklist: Recommended items to address in a clinical trial protocol and related documents.

## Data Availability

Data and protocol will be available from funder.

## Abbreviations

DSM-V: Diagnostic and Statistical Manual of Mental Disorders, Fifth Edition
IE: Independent evaluator
TAPES: Teacher Anxiety Program for Elementary Students
TAT: Teacher Anxiety Training
